# Multi-scale dynamics by adjusting the leaking rate to enhance the performance of deep echo state networks

**DOI:** 10.3389/frai.2024.1397915

**Published:** 2024-07-16

**Authors:** Shuichi Inoue, Sou Nobukawa, Haruhiko Nishimura, Eiji Watanabe, Teijiro Isokawa

**Affiliations:** ^1^Graduate School of Information and Computer Science, Chiba Institute of Technology, Narashino, Japan; ^2^LY Corporation, Chiyoda-ku, Japan; ^3^Department of Computer Science, Chiba Institute of Technology, Narashino, Japan; ^4^Research Center for Mathematical Engineering, Chiba Institute of Technology, Narashino, Japan; ^5^Department of Preventive Intervention for Psychiatric Disorders, National Institute of Mental Health, National Center of Neurology and Psychiatry, Kodaira, Japan; ^6^Faculty of Informatics, Yamato University, Osaka, Japan; ^7^Laboratory of Neurophysiology, National Institute for Basic Biology, Okazaki, Japan; ^8^Department of Basic Biology, The Graduate University for Advanced Studies, Hayama, Japan; ^9^Graduate School of Engineering, University of Hyogo, Himeji, Japan

**Keywords:** multi-scale dynamics, machine learning, reservoir computing, echo state network, deep echo state network

## Abstract

**Introduction:**

The deep echo state network (Deep-ESN) architecture, which comprises a multi-layered reservoir layer, exhibits superior performance compared to conventional echo state networks (ESNs) owing to the divergent layer-specific time-scale responses in the Deep-ESN. Although researchers have attempted to use experimental trial-and-error grid searches and Bayesian optimization methods to adjust the hyperparameters, suitable guidelines for setting hyperparameters to adjust the time scale of the dynamics in each layer from the perspective of dynamical characteristics have not been established. In this context, we hypothesized that evaluating the dependence of the multi-time-scale dynamical response on the leaking rate as a typical hyperparameter of the time scale in each neuron would help to achieve a guideline for optimizing the hyperparameters of the Deep-ESN.

**Method:**

First, we set several leaking rates for each layer of the Deep-ESN and performed multi-scale entropy (MSCE) analysis to analyze the impact of the leaking rate on the dynamics in each layer. Second, we performed layer-by-layer cross-correlation analysis between adjacent layers to elucidate the structural mechanisms to enhance the performance.

**Results:**

As a result, an optimum task-specific leaking rate value for producing layer-specific multi-time-scale responses and a queue structure with layer-to-layer signal transmission delays for retaining past applied input enhance the Deep-ESN prediction performance.

**Discussion:**

These findings can help to establish ideal design guidelines for setting the hyperparameters of Deep-ESNs.

## 1 Introduction

Reservoir computing, which is a branch of recurrent neural networks (RNNs), has garnered significant interest in terms of machine-learning applications (Lukoševičius and Jaeger, 2009; Tanaka et al., 2019; Gallicchio and Micheli, 2021). A neural network for reservoir computing consists of three layers: an input layer, a reservoir layer, and an output layer (Jaeger, 2001; Lukoševičius and Jaeger, 2009). In reservoir computing, the input time-series data are transformed into spatio-temporal patterns in the reservoir layer. The responses of individual neurons act as a kernel for these transformed patterns, representing the desired output signal, which is consequently used for time-series prediction and classification (Jaeger, 2001; Gallicchio and Micheli, 2021).

**Figure 1:**
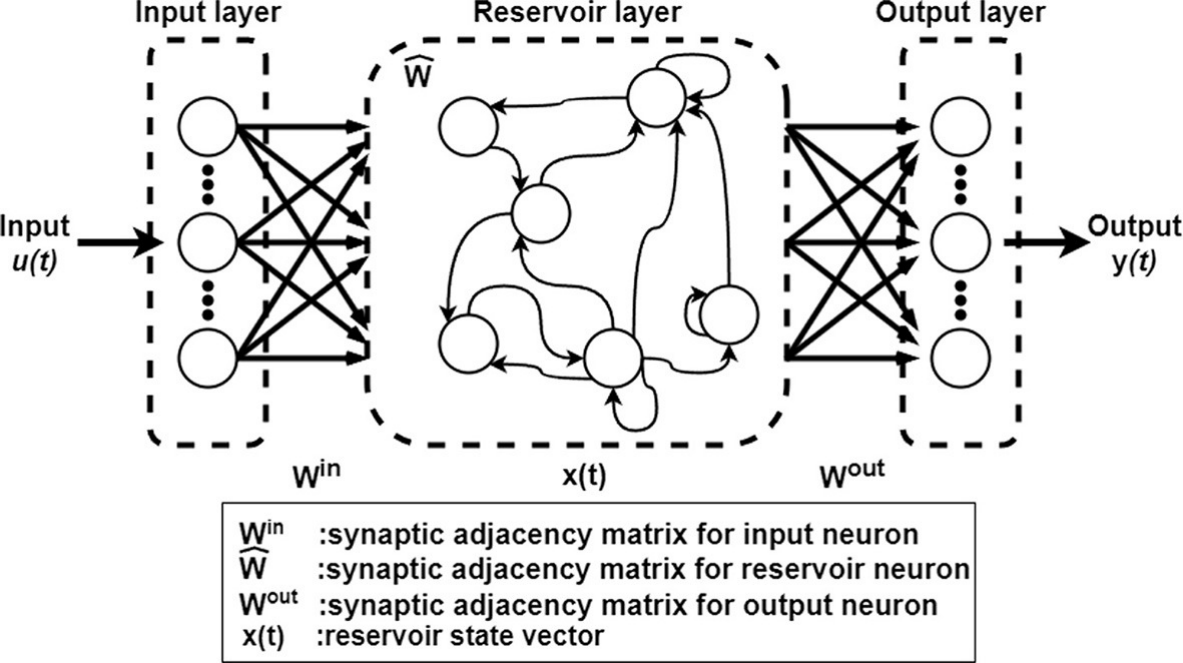
Architecture of ESN.

The echo state network (ESN), which is a representative model in reservoir computing, operates based on the response of the firing rate (Jaeger, 2007). In an ESN, as depicted in Figure 1, the synaptic connections of the reservoir weights are fixed, and only the synaptic connections of the output weight matrix are adjusted in the learning process (Tanaka et al., 2019). This architecture differs from that of other RNNs, wherein all synaptic connections within the network undergo updates during learning (Williams and Zipser, 1989). Thus, ESNs are more learning efficient (Werbos, 1990) than the widely utilized long-short-term memory model, despite exhibiting lower accuracy (Salehinejad et al., 2017; Gallicchio et al., 2018). Such efficient learning architectures may offer the potential for applications in areas that are characterized by resource-limited hardware, such as edge devices (Tanaka et al., 2019; Sakemi et al., 2024).

**Figure 2:**
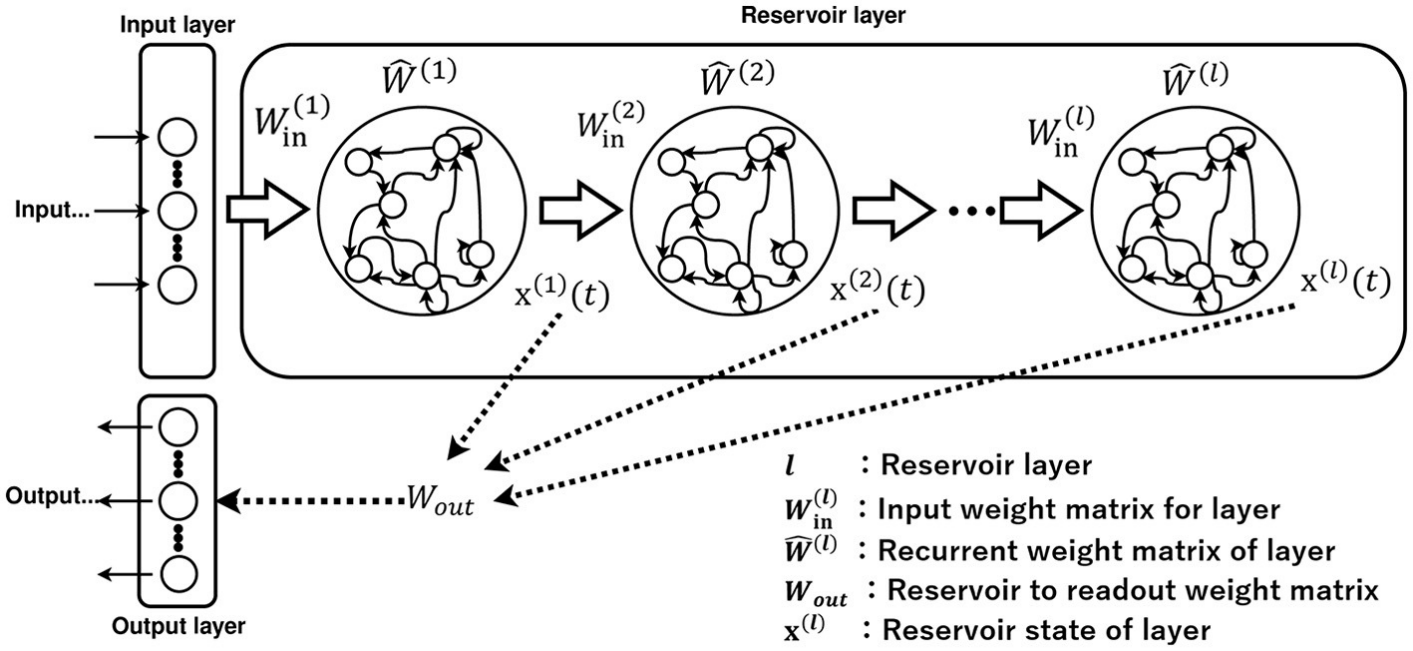
Architecture of Deep-ESN.

The deep echo state network (Deep-ESN) model, which possesses the multi-layered reservoir structure illustrated in Figure 2, has also been proposed. This model performs better than the conventional ESN, which consists of a single-layered reservoir (Deng et al., 2012; Gallicchio et al., 2017; Gallicchio and Micheli, 2021). The divergent responses in each layer of the Deep-ESN, which exhibits multiple time-scale dynamics, enhance the memory capacity and feature representation compared to its single-layered counterpart (Malik et al., 2016; Gallicchio et al., 2017; Tchakoucht and Ezziyyani, 2018; Gallicchio and Micheli, 2019; Long et al., 2019; Kanda and Nobukawa, 2022). These advantages of the Deep-ESN may enable applications in tasks involving nonlinear dynamic signals that exhibit multi-time-scale behaviors in various types of systems, such as biological systems, power systems, and financial markets (Venkatasubramanian et al., 1995; Costa et al., 2002; Bhandari, 2017; Chen and Shang, 2020; Yan and He, 2021).

The adjustment of numerous hyperparameters in the Deep-ESN is often based on experimental measurements, trial-and-error grid searches, and Bayesian optimization methods (Adeleke, 2019; Lukoševičius and Uselis, 2019; Bai et al., 2023; Viehweg et al., 2023). In terms of Bayesian optimization in Deep-ESNs, emphasis is mainly placed on performance, and the analysis of the reservoir dynamics and mechanisms of functionality enhancement is often overlooked; consequently, no specific design guidelines on the dynamics have been presented (Bai et al., 2023). In terms of hyperparameters that adjust the time scale of each layer, several studies have applied scaling methods to the input weight matrix *W*^(*l*)^, as illustrated in Figure 2 (Kanda and Nobukawa, 2022), to achieve the establishment of guidelines. By integrating this method, the signal strength between layers decreases as the depth increases, which results in a layer-specific time-scale response for each layer (Kanda and Nobukawa, 2022). This characteristic has been revealed by analyzing the layer dynamics using the multi-scale entropy (MSCE) method (Humeau-Heurtier, 2015; Kanda and Nobukawa, 2022). However, this approach, in which the input weights are scaled, has a major drawback. The signal strength in the reservoir layer diminishes quickly owing to the small scaling rate between layers. In addition, the leaking rate is a hyperparameter that influences the temporal history effect of the dynamics in **x**^(*l*)^(*t*) of Figure 2 (Jaeger et al., 2007; Schrauwen et al., 2007). Essentially, the leaking rate adjusts the decay factor of the dynamics in each neuron (Jaeger et al., 2007; Schrauwen et al., 2007). Therefore, the method for adjusting the leaking rate is another suitable candidate for achieving the layer-specific dynamical response in the Deep-ESN.

In this context, we hypothesized that evaluating the dependence of the dynamical response in the multi-layered reservoir in terms of adjusting the leaking rate would provide insights into achieving a guideline for optimizing the hyperparameters of the Deep-ESN. For the preliminary investigation, we set the same leaking rate for each layer of the Deep-ESN and performed an MSCE analysis to investigate the impact of the leaking rate on the dynamics in each layer. The results confirmed that each layer of the Deep-ESN generates dynamics at different time scales, which induces a queue-like property whereby the delay response is preserved by the hierarchical structure (Inoue et al., 2023). However, the performance tendencies for more diverse time-series prediction tasks remain unclear; an evaluation in the case of setting heterogeneous leaking rates in the multi-layered reservoir has not been conducted. Therefore, in this study, based on the preliminary outcomes of a previous study (Inoue et al., 2023), we further revealed these points. Specifically, Deep-ESNs with homogenous and heterogeneous leaking rates for each layer were used to perform and evaluate a time-series prediction task using three time-series signals: the Lorenz, Rössler, and Mackey–Glass models. Furthermore, we performed layer-by-layer MSCE and cross-correlation analyses between adjacent layers to elucidate the mechanisms behind the functional enhancement achieved through the leaking rates.

## 2 Materials and methods

### 2.1 ESN

Figure 1 shows the architecture of the ESN. The input signal is defined as $\text{u}\left(t\right)\in {\mathbb{R}}^{N_{u}}$ with *N*_*u*_-dimensional inputs. The reservoir state is defined by $\text{x}\left(t\right)\in {\mathbb{R}}^{N_{x}}$, where *N*_*x*_ is the number of neurons in the reservoir layer. The reservoir state **x**(*t*) is defined by


$$\begin{array}{l} \text{x}\left(t\right)=\left(1-a\right)\text{x}\left(t-1\right)+a\tanh\left({\text{W}}_{\text{in}}\text{u}\left(t\right)+\hat{\text{W}}\text{x}\left(t-1\right)\right), \end{array}$$

where *a*∈[0, 1] is the leaking rate and ${\text{W}}_{\text{in}}\in {\mathbb{R}}^{N_{x}\times N_{u}}$ is the input weight matrix. Each component of **W**_in_ is represented by a uniform random value, the range of which is [−*s*_in_, *s*_in_]. $\hat{\text{W}}\in {\mathbb{R}}^{N_{x}\times N_{x}}$ is the recurrent synaptic weight matrix, which is a random matrix with uniform random numbers, and its spectral radius is set to ρ. The output of the ESN at time *t* is determined using


$$\begin{array}{l} \text{y}\left(t\right)={\text{W}}_{\text{out}}\text{x}\left(t\right), \end{array}$$

where ${\text{W}}_{\text{out}}\in {\mathbb{R}}^{N_{y}\times N_{x}}$ is defined as the output weight matrix and $\text{y}\left(t\right)\in {\mathbb{R}}^{N_{y}}$ is the *N*_*y*_-dimensional output. The initial value of **W**_out_ is a random matrix of uniform random numbers.

### 2.2 Deep-ESN

The multi-layered Deep-ESN is constructed based on the single-layered ESN; Figure 2 exhibits a diagram of the Deep-ESN (Deng et al., 2012; Gallicchio et al., 2017; Gallicchio and Micheli, 2021). The only difference from the ESN is that the reservoir layer is multi-layered. The reservoir state vector of the Deep-ESN is defined by


$$\begin{array}{l} x^{\left(l\right)}(t)=(1-a^{\left(l\right)})x^{\left(l\right)}(t-1)+a^{\left(l\right)}\tanh(W_{\text{in}}^{\left(l\right)}i^{\left(l\right)}(t)\\ \;+\theta^{\left(l\right)}+{\hat{W}}^{\left(l\right)}x^{\left(l\right)}(t-1)), \end{array}$$

where variables consisting of *l* refer to *l*-layer parameters. ${\text{θ}}^{\left(l\right)}\in {\mathbb{R}}^{N_{x}}$ is the bias in the reservoir coupling weight matrix. ${\text{W}}_{\text{in}}^{\left(l\right)}\in {\mathbb{R}}^{N_{x}\times N_{u}}$ is the input weight matrix for each layer, and ${\hat{\text{W}}}^{\left(l\right)}\in {\mathbb{R}}^{N_{x}\times N_{x}}$ is the recurrent weight matrix for each layer. The dynamics of each reservoir layer can be defined as $\bar{x^{\left(l\right)}\left(t\right)}$ by averaging the reservoir state vector **x**^(*l*)^(*t*) over all neurons. In addition, **i**^(*l*)^(*t*) represents the input to the *l*-th layer in the Deep-ESN, which is expressed as


$$i^{\left(l\right)}(t)=\{\begin{array}{ll} u(t) & \text{if }l=1,\\ x^{\left(l-1\right)}(t) & \text{if }l>1. \end{array}$$

The output of the Deep-ESN at time *t* is determined by


$$y(t)=W_{\text{out}}{\left[x^{\left(1\right)}(t)\;\;x^{\left(2\right)}(t)\;\;...\;\;x^{\left(N_{L}\right)}(t)\right]}^{⊤}+\theta_{\text{out}},$$

where *N*_*L*_ is defined as the total number of reservoir layers and $\text{y}\left(t\right)\in {\mathbb{R}}^{N_{y}}$ is the output in *N*_*y*_ dimensions. ${\text{W}}_{\text{out}}\in {\mathbb{R}}^{N_{y}\times N_{L}N_{x}}$ is the output weight matrix. The bias in the output layer is set to $\theta_{\text{out}}={\left[1,1,...,1\right]}^{⊤}$.

In this study, the total number of layers was set to *N*_*L*_ = 10, the number of neurons in each layer was set to *N*_*x*_ = 100, the scaling parameter of the input weight matrix **W**_in_ was set to *s*_in_ = 1, the spectral radius was set to ρ = 1.0, 0.9, 0.8, and ridge regression was used as the learning algorithm. For the settings of the leaking rate *a*^(*l*)^ in this experiment, a homogeneous model with the same leaking rate in all layers and a heterogeneous model with different leaking rates in each layer were used. The leaking rate *a*^(*l*)^ of the homogeneous model was set to be *a*^(*l*)^ = 1.0, 0.9, ..., 0.1 in all layers. In the heterogeneous model, the leaking rate *a*^(*l*)^ was set to decrease incrementally by 0.1 across each layer, starting from 1.0 and decreasing to 0.1. For simplicity, the same values were set for the hyperparameters *N*_*L*_, *N*_*x*_, *s*_in_, ρ, and *a*^(*l*)^ for each layer in the reservoir. In addition, **W**_in_, ${\hat{\text{W}}}^{\left(l\right)}$, **W**_out_, and **x**(0) were initialized with different random seeds for each trial. The seed values were changed during the execution of the time-series prediction task, and 100 trials were performed.

### 2.3 Time-series prediction task

In terms of the impact of the leaking rate on the performance, the Mackey–Glass, Lorenz, and Rössler equations were prepared as time-series signals with different dynamical characteristics. In this study, homogeneous and heterogeneous models were used in the time-series prediction task, which was evaluated by predicting the five-step-ahead value from the current input. The input signal was continuously applied to the models. Each task involved 100 trials with different initial values. The normalized root mean square error (NRMSE) was used to evaluate the prediction accuracy for each task in the homogeneous and heterogeneous models.

#### 2.3.1 Mackey–Glass equation

For the time-series prediction, time series were generated from the Mackey–Glass equation (Glass and Mackey, 2010):


$$\begin{array}{l} \begin{array}{c} \frac{dx_{\text{mg}}}{dt}=\frac{0.2x_{\text{mg}}\left(t-\tau \right)}{1+x_{\text{mg}}{\left(t-\tau \right)}^{10}}-0.1x_{\text{mg}}\left(t\right), \end{array} \end{array}$$

where τ is a constant representing the delay. In this study, under the condition of τ = 32, 64, and 128, the solution was obtained using the fourth-order Runge–Kutta method, and the trajectories were sampled in a time window of Δ*t* = 10.

#### 2.3.2 Lorenz equation

The Lorenz equation is represented by a system of nonlinear differential equations of the form (Manneville and Pomeau, 1979)


$$\begin{array}{l} \frac{dx_{\text{l}}}{dt}=\sigma (y_{\text{l}}-x_{\text{l}}),\\ \frac{dy_{\text{l}}}{dt}=x_{\text{l}}(\rho -z_{\text{l}})-y_{\text{l}},\\ \frac{dz_{\text{l}}}{dt}=x_{\text{l}}y_{\text{l}}-\beta z_{\text{l}}. \end{array}$$

The parameters for the Lorenz equation were σ = 10, *r* = 28, and *b* = 8/3. These values are known to exhibit chaotic behavior. In this study, the solution was obtained using the fourth-order Runge–Kutta method, and the trajectories were sampled in the time window Δ*t* = 0.02.

#### 2.3.3 Rössler equation

The Rössler system, which is a nonlinear dynamical system (Rössler, 1983), was adopted to generate chaotic time-series data. The system is defined by the following set of three nonlinear ordinary differential equations:


$$\begin{array}{ll} \frac{dx_{\text{r}}}{dt} & =-y_{\text{r}}-z_{\text{r}}, \end{array}$$

$$\begin{array}{ll} \frac{dy_{\text{r}}}{dt} & =x_{\text{r}}+ay_{\text{r}},\\ \frac{dz_{\text{r}}}{dt} & =b+z_{\text{r}}\left(x_{\text{r}}-c\right). \end{array}$$

In these equations, *x*_r_, *y*_r_, and *z*_r_ represent the system states. The parameters *a*, *b*, and *c* directly affect the system's behavior. For our experiments, the parameters were set to *a* = 0.2, *b* = 0.2, and *c* = 5.7. In this study, the solution was obtained using the fourth-order Runge–Kutta method, and the trajectories were sampled in the time window Δ*t* = 0.02.

### 2.4 MSCE analysis

MSCE analysis is a method for performing coarse-graining of the time series of interest and quantitatively evaluating the complexity across multiple time scales (Humeau-Heurtier, 2015). As an analytical procedure, the first step is to coarse-grain the dynamics of each reservoir layer $\bar{x^{\left(l\right)}\left(t\right)}$ with a time-scale factor τ_*s*_, using


$$\begin{array}{l} z_{j}^{\left(\tau_{s},l\right)}=\left(\frac{1}{\tau_{s}}\right)\overset{j\tau_{s}}{\underset{i=\left(j-1\right)\tau_{s}+1}{\sum }}\bar{x^{\left(l\right)}\left(i\right)},\;(1\leq j\leq \frac{N}{\tau_{s}}). \end{array}$$

In the case of τ_*s*_ = 1, the original time series is coarse-grained to longer-scale dynamics as τ_*s*_ increases. At each time scale τ_*s*_ and layer *l*, the complexity of the coarse-grained time series is then quantified by the sample entropy (SampEn). SampEn is given by


$$\begin{array}{l} \text{SampEn}\left(r,m,N\right)=-\log\frac{U_{m+1}\left(r\right)}{U_{m}\left(r\right)}, \end{array}$$

where *U*_*m*_(*r*) represents the probability of being $|{\text{z}}_{i}^{m}-{\text{z}}_{j}^{m}|<r\left(i\neq j,i,j=1,2,...\right)$ and ${\text{z}}_{i}^{m}$ represents the *m*-dimensional vector ${\text{z}}_{i}^{m}=\left\{z_{i}^{\left(\tau_{s},l\right)},z_{i+1}^{\left(\tau_{s},l\right)},...,z_{i+m-1}^{\left(\tau_{s},l\right)}\right\}$. Thus, the complexity of the dynamics of each layer can be analyzed and evaluated from different time-scale perspectives.

### 2.5 NRMSE

The NRMSE is a statistical measure that is used to assess the accuracy of a model's predictions and is defined as follows:


$$\text{NRMSE}=\sqrt{\frac{\sum_{t=1}^{T}(y(t)-y_{d}(t){)}^{2}}{T\sigma^{2}(y_{d})}}.$$

In this study, the task inputs and outputs were one-dimensional. Therefore, *y*(*t*) is the output of the ESN in the case with *N*_*y*_ = 1 at time *t*, *y*_*d*_(*t*) is the teacher signal at time *t*, σ^2^(*y*(*t*)) is the variance of the teacher signal, and *T* is the evaluation period (number of data points). The NRMSE was evaluated among 100 trials with different initial conditions.

### 2.6 Cross-correlation analysis

Cross-correlation analysis, which evaluates the synchronization with a delay between two time-series signals, is extensively used for the signal transmission of neural activity in hierarchical brain networks (Adhikari et al., 2010; Dean and Dunsmuir, 2016). Thus, we adopted the cross-correlation analysis for the signal transmission in the Deep-ESN. We used the cross-correlation Corr(*k*) between the dynamics of the reservoir state at the *l*-th and *l*+1-th layers, i.e., the time-series $\bar{x^{\left(l\right)}\left(t\right)}$ and $\bar{x^{\left(l+1\right)}\left(t-k\right)}$, where *k* is the delay time and each time series is *z*-score transformed.

### 2.7 Experimental procedures

This study implemented the following procedures to explore parameters that demonstrate dynamics that can achieve high performance in Deep-ESNs: We initially employed MSCE analysis to identify the time-scale dependency of the dynamical responses in terms of complexity among the layers. This analysis aims to guide the optimization of Deep-ESNs for enhanced handling of time-series prediction tasks. After completing the MSCE analysis, we proceeded with the synchronization analysis to detect delays between adjacent layers. This sequential approach facilitates a comprehensive understanding of both the dynamics of each layer and the interactions between layers. Through these analyses, our focus shifted to establishing guidelines for setting hyperparameters, particularly in cases where previous studies on Deep-ESNs did not yield significant findings on the dynamical characteristics. These guidelines are designed to refine the time scale of the dynamics in Deep-ESNs based on the insights gained from our MSCE and cross-correlation analyses.

## 3 Results

### 3.1 Time-series prediction task

**Figure 3:**
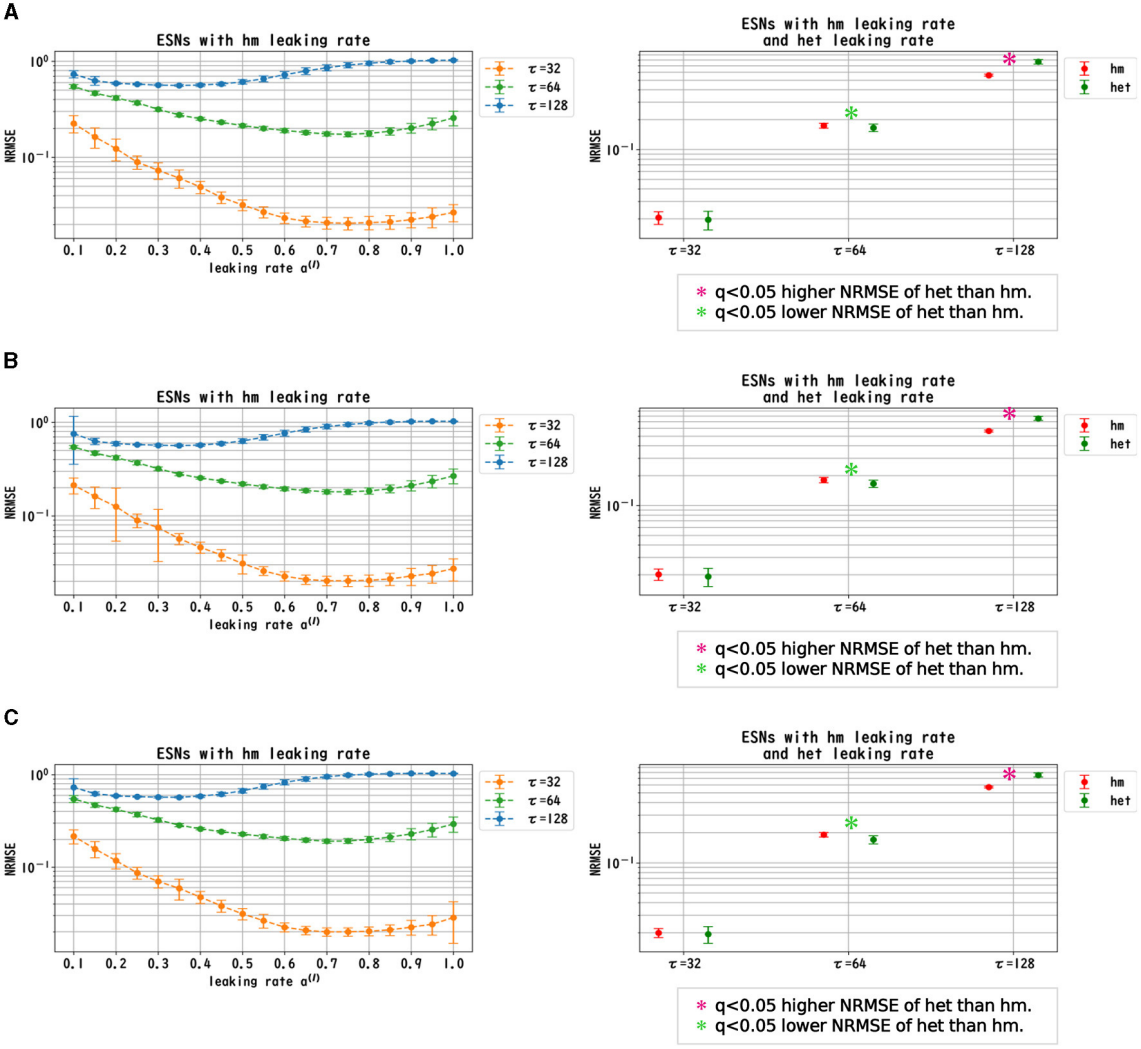
Prediction performance in the Mackey–Glass time series. (Left panel) Dependence of the performance of the Deep-ESN on leaking rates (*a*^(*l*)^ = 1.0, 0.9, ..., 0.1 for all layers) in the homogeneous (hm) model with spectral radii ρ = 1.0 **(A)**, 0.9 **(B)**, and 0.8 **(C)**. The profiles of the NRMSEs in all spectral radii exhibited a *U*-shape against the leaking rate, indicating the presence of an optimal leaking rate for the prediction tasks. (Right panel) NRMSEs for the most superior cases in the homogeneous model (corresponding to the cases with minimum NRMSEs presented in the left panel) and for the cases of the heterogeneous (het) model in which the leaking rate was set to decrease by 0.1 in each layer, from 1.0 to 0.1. The NRMSE of the heterogeneous model was low [based on the paired-*t* test using a Bonferroni false discovery rate with *q* < 0.05 (*p* < 0.05/9)] only for the Mackey–Glass time series (τ = 64) throughout all spectral radii.

**Figure 4:**
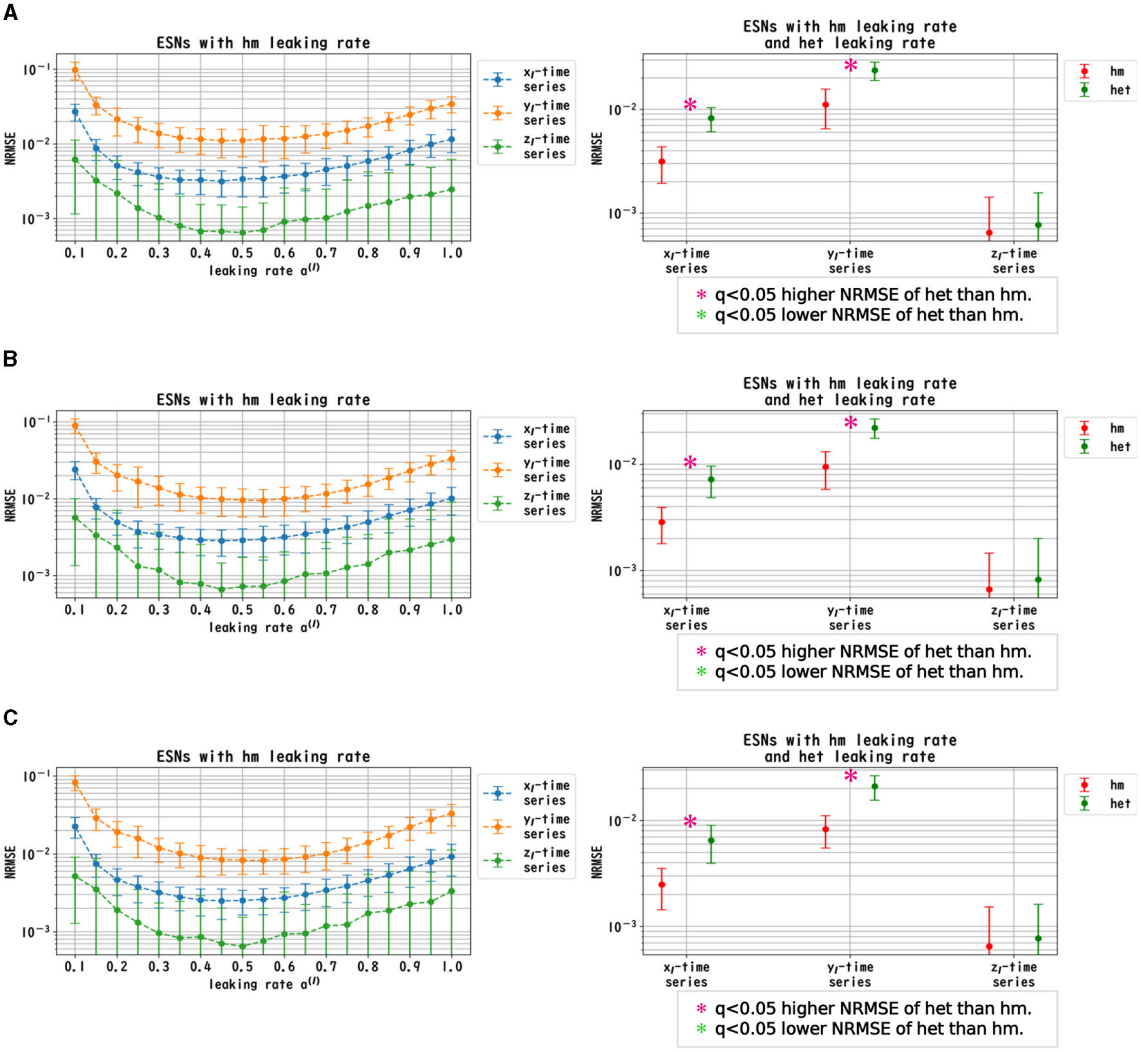
Prediction performance in the Lorenz time series. (Left panel) Dependence of the performance of the Deep-ESN on leaking rates [*a*^(*l*)^ = 1.0, 0.9, ..., 0.1 for all layers] in the homogeneous (hm) model with spectral radii ρ = 1.0 **(A)**, 0.9 **(B)**, and 0.8 **(C)**. The profiles of the NRMSEs in all spectral radii exhibited a *U*-shape in response to the leaking rate, indicating the presence of an optimal leaking rate for the prediction tasks. (Right panel) NRMSEs for the most superior cases in the homogeneous model (corresponding to the cases with the minimum NRMSEs highlighted in the left panel) and for the cases of the heterogeneous (het) model in which the leaking rate was set to decrease by 0.1 in each layer, from 1.0 to 0.1. The NRMSE of the homogeneous model was low [based on the paired-*t* test using a Bonferroni false discovery rate with *q* < 0.05 (*p* < 0.05/9)] for the Lorenz time series [*x*_l_(*t*) and *y*_l_(*t*)] throughout all spectral radii.

**Figure 5:**
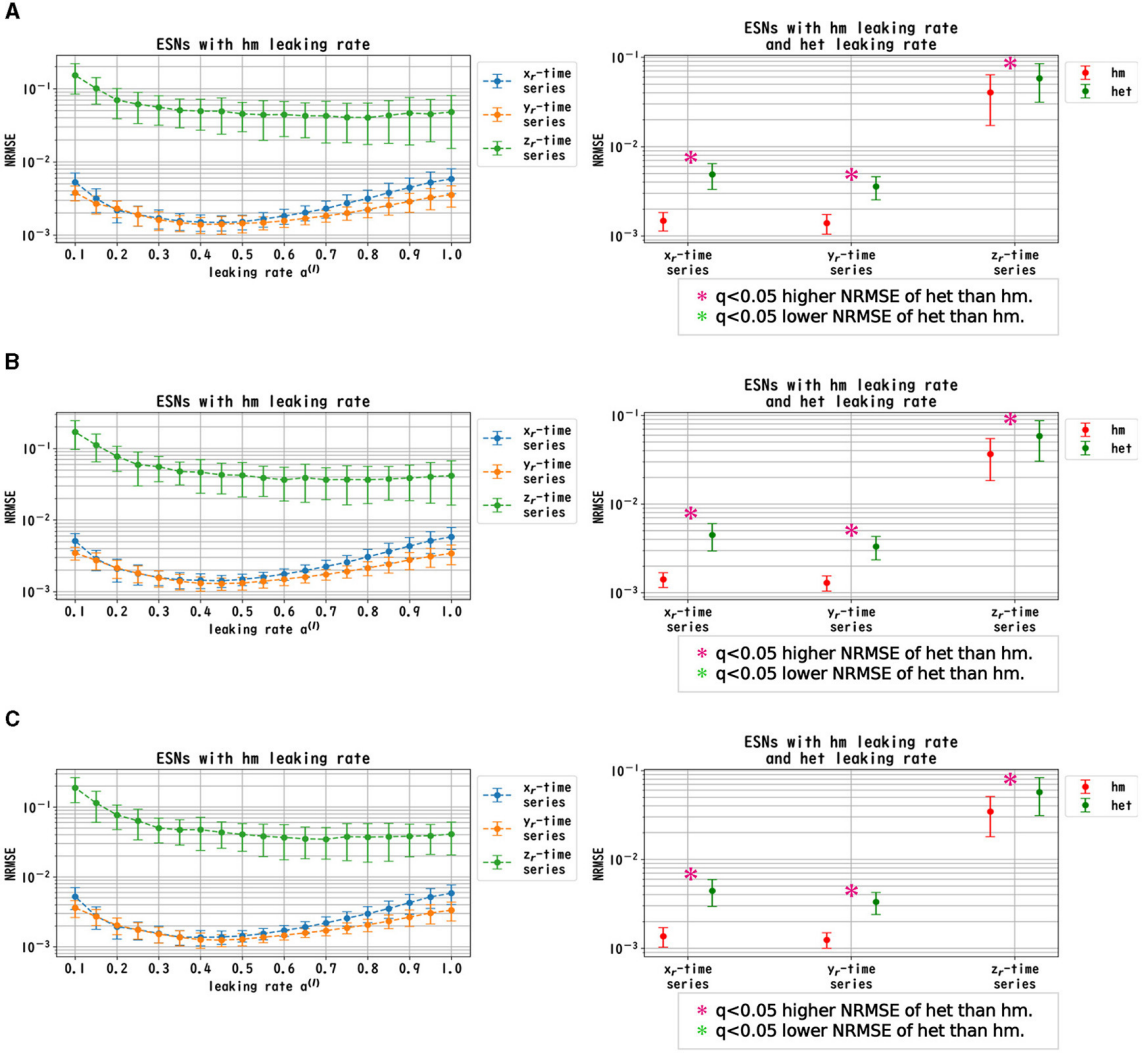
Prediction performance in the Rössler time series. (Left panel) Dependence of the performance of the Deep-ESN on leaking rates [*a*^(*l*)^ = 1.0, 0.9, ..., 0.1 for all layers] in the homogeneous (hm) model with spectral radii ρ = 1.0 **(A)**, 0.9 **(B)**, and 0.8 **(C)**. The profiles of the NRMSEs in all spectral radii exhibited a *U*-shape against the leaking rate, indicating the presence of an optimal leaking rate for the prediction tasks. (Right panel) NRMSEs for the most superior cases in the homogeneous model (corresponding to the cases with the minimum NRMSEs shown in the left panel) and for the cases of the heterogeneous (het) model in which the leaking rate was set to decrease by 0.1 in each layer, from 1.0 to 0.1. The NRMSE of the homogeneous model was low [based on the paired-*t* test using a Bonferroni false discovery rate with *q* < 0.05 (*p* < 0.05/9)] for the Rössler time series [*x*_l_(*t*), *y*_l_(*t*) and *z*_l_(*t*)] throughout all spectral radii.

We evaluated the dependence of the performance of Deep-ESNs on the leaking rates *a*^(*l*)^ in the time-series prediction tasks in the nonlinear dynamical signals of the Mackey–Glass, Lorenz, and Rössler equations. Figures 3–5 convey the results of the evaluation of the homogeneous and heterogeneous models in each time-series prediction task. In the homogeneous model, the leaking rate was set to *a*^(*l*)^ = 1.0, 0.9, ..., 0.1 for all layers. In the heterogeneous model, the leaking rate decreased by 0.1 in each layer, from 1.0 to 0.1. The dependences of the NRMSE on the leaking rate in the homogeneous model for the spectral radii ρ = 0.8, 0.9, and 1.0 are displayed in the left panels of Figures 3–5. The results demonstrate that the profiles of the NRMSEs in all tasks and the spectral radii exhibited a *U*-shape in response to the leaking rate, indicating the presence of an optimal leaking rate for the prediction tasks. Furthermore, the right panels of Figures 3–5 present a comparison of the NRMSEs of the most superior cases in the homogeneous model, which were obtained from the profile of the dependence on the leaking rate in the left panels, with the heterogeneous model leaking rate. The results reveal that the NRMSE of the heterogeneous model was significantly low (based on the paired-*t* test using a Bonferroni false discovery rate with *q* < 0.05) only for the Mackey–Glass time series (τ = 64) across all spectral radii. This tendency implied that the heterogeneous models could precisely respond to strong multi-temporal-scale dynamics, similar to the Mackey–Glass time series (τ = 64) that exhibited wide multi-temporal-scale components in this dynamics (see Section 1 of Supplementary material).

### 3.2 MSCE analysis

**Figure 6:**
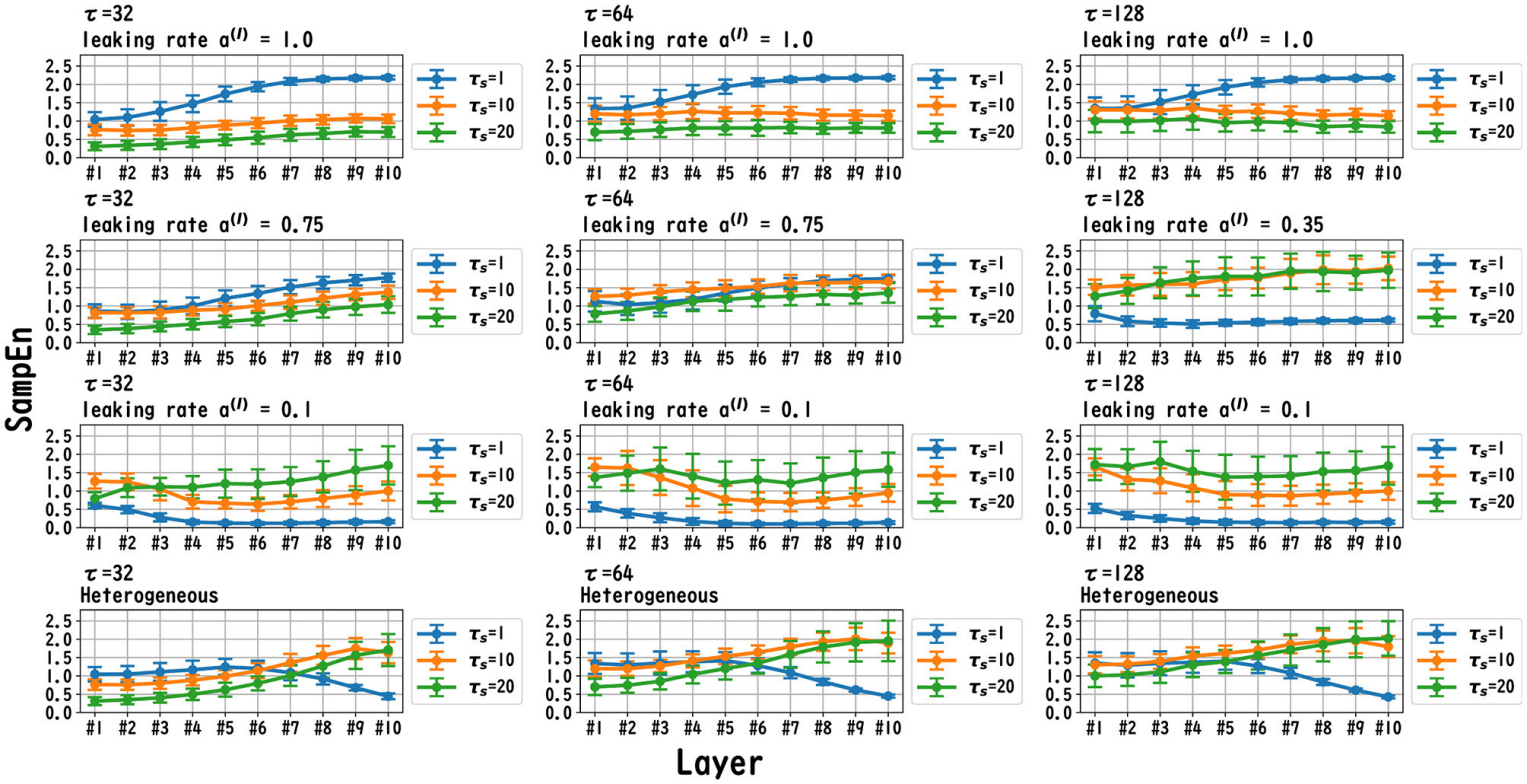
MSCE analysis of reservoir dynamics (temporal scale τ_*s*_ = 1, 10, 20) with spectral radius ρ = 1.0 in the case of the Mackey–Glass task. The first, second, and third columns list the cases with Mackey–Glass delay time constants τ = 32, 64, and 128, respectively. The first, second, and third columns correspond to the maximum value of the leaking rate [*a*^(*l*)^ = 1.0], the leaking rate when the NRMSE was the best, and the minimum leaking rate [*a*^(*l*)^ = 0.1], respectively. In the case of the homogeneous model, a trend toward reduced complexity on the fast time scale (τ_*s*_ = 1) was observed as the leaking rate was reduced. The complexity on the slow time scales (τ_*s*_ = 10, 20) tended to vary across layers or to be almost constant depending on the time scale of the task and leaking rate. In the heterogeneous model case, the complexity on the fast time scale (τ_*s*_ = 1) tended to decrease according to the layer depth. The complexity on the slow time scale (τ_*s*_ = 10, 20) tended to increase layer by layer.

**Figure 7:**
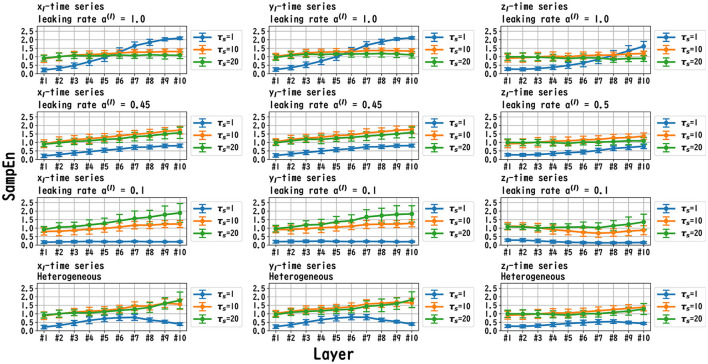
MSCE analysis of reservoir dynamics (temporal scale τ_*s*_ = 1, 10, 20) with spectral radius ρ = 1.0 in the case of the Lorenz task. The first, second, and third columns display the cases with Lorenz tasks *x*_l_(*t*), *y*_l_(*t*), and *z*_l_(*t*), respectively. The first, second, and third columns correspond to the maximum value of the leaking rate [*a*^(*l*)^ = 1.0], the leaking rate when the NRMSE was the best, and the minimum value of the leaking rate [*a*^(*l*)^ = 0.1], respectively. In the case of the homogeneous model, a trend toward reduced complexity on the fast time scale (τ_*s*_ = 1) was observed as the leaking rate was reduced. The complexity on the slow time scales (τ_*s*_ = 10, 20) tended to vary across layers or to be almost constant, depending on *x*_*l*_/*y*_*l*_/*z*_*l*_ and the leaking rate. In the heterogeneous model case, the complexity on the fast time scale (τ_*s*_ = 1) tended to decrease according to the layer depth. The complexity on the slow time scales (τ_*s*_ = 10, 20) tended to increase layer by layer.

**Figure 8:**
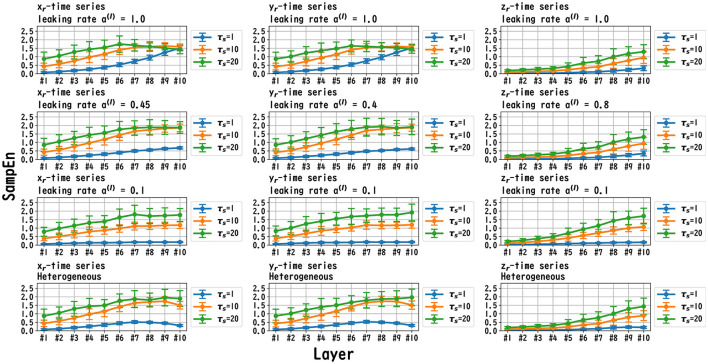
MSCE analysis of reservoir dynamics (temporal scale τ_*s*_ = 1, 10, 20) with spectral radius ρ = 1.0 in the case of the Rössler task. The first, second, and third columns convey the cases with Rössler tasks *x*_r_(*t*), *y*_r_(*t*), and *z*_r_(*t*), respectively. The first, second, and third columns correspond to the maximum value of the leaking rate [*a*^(*l*)^ = 1.0], the leaking rate when the NRMSE was the best, and the minimum value of the leaking rate [*a*^(*l*)^ = 0.1], respectively. In the homogeneous case with task *x*_r_(*t*), *y*_r_(*t*), in the case with a high leaking rate, the complexity of the fast time scale (τ_*s*_ = 1) increased with the layer depths. The complexity on the slow time scales (τ_*s*_ = 10, 20) tended to vary across layers or to be almost constant, depending on *x*_*r*_/*y*_*r*_/*z*_*r*_ and the leaking rate. In the heterogeneous model case with *x*_r_(*t*) and *y*_r_(*t*), the complexity on the fast time scales (τ_*s*_ = 1) tended to decrease with the layer depth. The complexity on the slow time scales (τ_*s*_ = 10, 20) tended to increase layer by layer.

MSCE analysis was used to investigate the behavior of the dynamics of each layer of the reservoir. Figures 6–8 exhibit the results of the MSCE analysis for the Mackey–Glass, Lorenz, and Rössler tasks, respectively. The temporal scales τ_*s*_ were set to 1, 10, and 20. In the case of the homogeneous model, a trend toward reduced complexity on the fast time scale (τ_*s*_ = 1) was observed as the leaking rate was reduced. The complexity with slow time scales (τ_*s*_ = 10, 20) may vary across layers or be almost constant, depending on the time scale of the task and leaking rate. In the heterogeneous model case, the complexity on the fast time scale (τ_*s*_ = 1) tended to decrease with the layer depth. The complexity on the slow time scales (τ_*s*_ = 10, 20) tended to increase layer by layer.

### 3.3 Cross-correlation analysis

**Figure 9:**
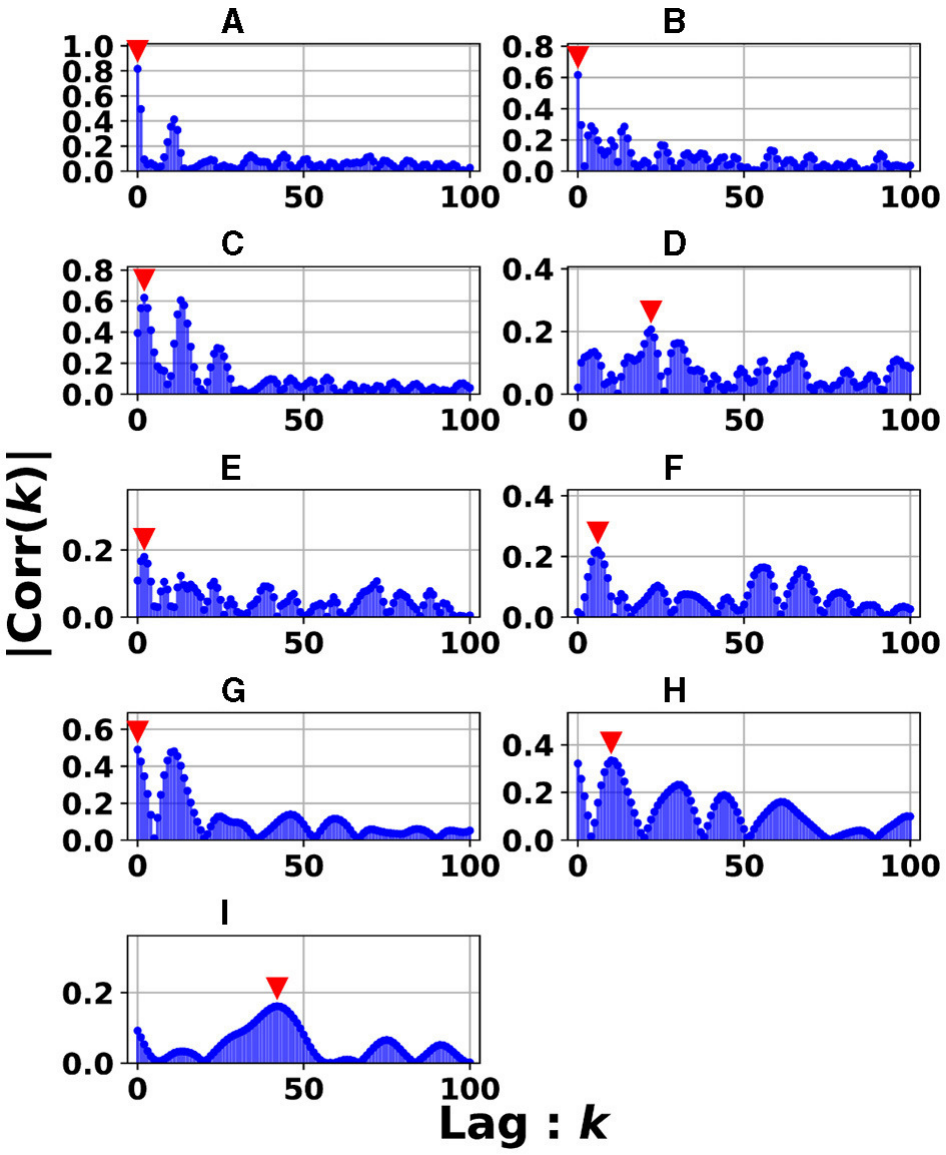
Absolute values of cross-correlation for the dynamics of the reservoir states between adjacent reservoir layers (*l*-th and *l*+1-th layers): |Corr(*k*)| in the case with the heterogeneous model (spectral radius ρ = 1.0) for the Mackey–Glass (τ = 64) task. This setting corresponds to the highest accuracy in Figure 3A for the Mackey–Glass (τ = 64) task. Lag *k* where the maximized |Corr(*k*)| was achieved (represented by the red arrow), expresses the signal transmission delay from the *l*-th to *l*+1-th layers. In panels **(C–I)**, the peaks are *k*≥1, indicating that the reservoir dynamics were delayed between layers. **(A)** #1 & #2. **(B)** #2 & #3. **(C)** #3 & #4. **(D)** #4 & #5. **(E)** #5 & #6. **(F)** #6 & #7. **(G)** #7 & #8. **(H)** #8 & #9. **(I)** #9 & #10.

**Figure 10:**
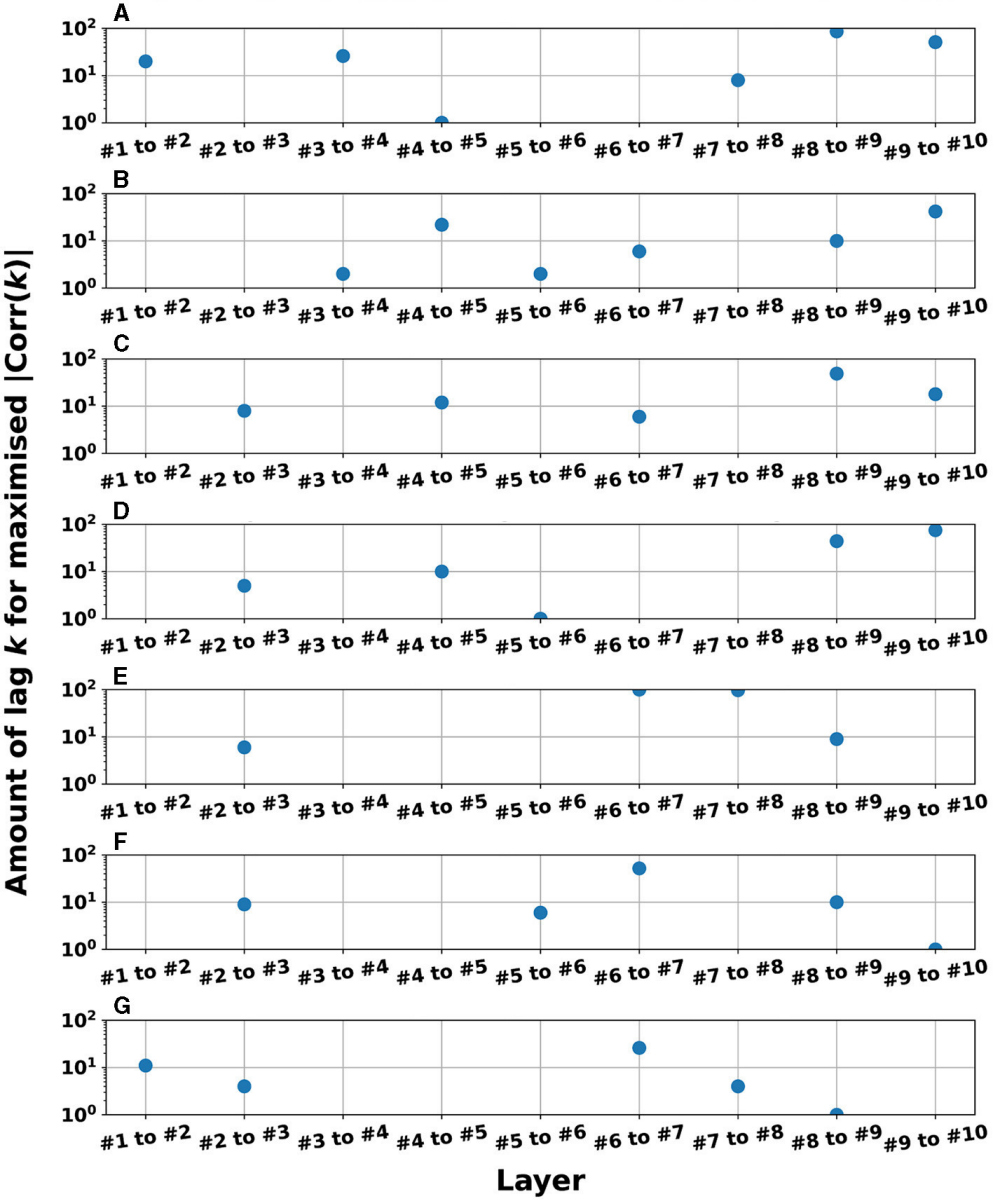
Lag *k*-values where |Corr(*k*)| was maximized for adjacent pair-wise layers in the cases with parameter settings (homogeneous/heterogeneous models) to achieve the superior NRMSE in the time-series prediction task (corresponding to the right panel of A in Figures 3–5). The values are not plotted when the reservoir dynamics of the *l*-th and *l*+1-th layers were zero-lag synchronized (the peak of correlation was *k* = 0) but plotted when the *l*+1-th layer was delayed against the *l*-th layer (the peak of correlation was *k*≥1). The presence of between four and six plot points in each subfigure confirms that reservoir dynamics delays occurred in the *l*-th and *l*+1-th layers. **(A)** Mackey-Glass (τ = 128), Homogeneous model, leaking rate α= 0.35. **(B)** Mackey-Glass (τ = 64), Homogeneous model. **(C)** Lorenz (x-time series), Homogeneous model, leaking rate α= 0.45. **(D)** Lorenz (y-time series), Homogeneous model, leaking rate α= 0.45. **(E)** Rössler (x-time series), Homogeneous model, leaking rate α= 0.45. **(F)** Rössler (y-time series), Homogeneous model, leaking rate α= 0.4. **(G)** Rössler (z-time series), Homogeneous model, leaking rate α= 0.8.

The signal transmissions in the dynamics of the reservoir state among layers were evaluated using cross-correlation analysis. Figure 9 depicts the dynamics of the reservoir states between adjacent reservoir layers (the *l*-th and *l*+1-th layers): |Corr(*k*)| in the case of the heterogeneous model (spectral radius ρ = 1.0) for the Mackey–Glass (τ = 64) task. This setting corresponds to the highest accuracy in Figure 3A for the Mackey–Glass (τ = 64) task. |Corr(*k*)| was maximized at the positive lag (*k*≥0) in the specific between layers (#3&#4, #4&#5, #5&#6, #6&#7, #8&#9, #9&#10), i.e., delays in signal transmission occurred from the *l*-th to *l*+1-th layers. To evaluate this tendency against the different tasks used in this study, Figure 10 presents the *k*-values where |Corr(*k*)| was maximized for adjacent pair-wise layers. The results demonstrate that a major part of the pair-wise layers in all tasks exhibited positive *k*-values (≥1); that is, signal transmission delays occurred between adjacent layers. This helps to retain past information. In addition to cross-correlation, it is necessary to evaluate synchronization with delays in systems involving nonlinear dynamics. Therefore, we analyzed the synchronization with delays between the time-series of the reservoir state at the *l*-th and *l*+1-th layers using mutual information under the conditions presented in Figure 9 (see Section 3 in Supplementary material). The results indicate that similar to the findings of the cross-correlation, the mutual information peaked at a positive lag (*k*>0), specifically between layers. This indicates that signal transmission delays also occurred from the *l*-th to *l*+1-th layer, considering the nonlinear relationships between the behaviors across layers. In addition, according to the analysis of the output weight matrix **W**_out_ in the readout, the contributions to the prediction tasks, which were facilitated by these multi-scale behaviors and layer-to-layer delays, were distributed among the layers (see Section 4 in Supplementary material).

## 4 Discussion

In this section, we first recapitulate the key findings derived from the results. Our investigation was primarily aimed at understanding the mechanism of the Deep-ESN function enhancement and guidelines by adjusting the leaking rate *a*^(*l*)^. In three different experiments, we validated our hypothesis that the evaluation of the dependence of the dynamical response in the multi-layered reservoir on adjusting the leaking rate would provide insights into achieving a guideline for optimizing the hyperparameters of the Deep-ESN. Specifically, the first experiment (time-series prediction tasks) with the homogeneous model showed that the profiles of the MSCE in all tasks and the spectral radii exhibited a *U*-shape against the leaking rate, indicating an optimal leaking rate for each prediction task. The heterogeneous model was effective for the Mackey–Glass time series (τ = 64), which contains wide multi-temporal-scale components in its dynamics. The second experiment (MSCE analysis) with the homogeneous model indicated that the complexity associated with fast time scales tended to decrease with the leaking rate, while that of the slow time scales tended to vary or remain almost constant according to the number of layers, depending on the task time scale and leaking rate. In the heterogeneous models, the fast time-scale complexity tended to decrease with layer depth, while the slow time-scale complexity tended to increase layer by layer. Finally, the third experiment (cross-correlation analysis) with homogeneous and heterogeneous models demonstrated the layer-to-layer signal transmission delay in all tasks, which helps to retain past information.

We first discuss the reasons for the presence of an optimum leaking rate (see right panel of Figures 3–5). In single neural dynamics, as the leaking rate increases, the dynamics of the neuron become faster. Based on this effect, the complexity of fast-scale dynamics in the case with a large leaking rate increases further, especially in deep layers, through the multiple-layered propagation (see the tendency of SampEn when increasing the leaking rate in the case with τ_*s*_ = 1 in Figures 6–8). This tendency was observed in the homogeneous case with a large leaking rate and in the heterogeneous case. Meanwhile, the complexity at slow time scales exhibited a diverse layer-specific SampEn profile, depending on the leaking rate and task (see the SampEn of τ_*s*_ = 10, 20 in Figures 6–8). This tendency may be attributed to the complex interactions in layer-to-layer signal propagation. In general, to achieve high ESN performance, the representation of complex desired signals requires the combination of diverse time-scale dynamical responses in the readout (Tanaka et al., 2022). The layer-specific time-scale dynamic response obtained using the Deep-ESN can satisfy this requirement, and this can be achieved by adjusting the leaking rate.

Next, we discuss the structural effectiveness of the Deep-ESN. The hierarchical structure causes delays in the layer-to-layer signal transmission (see Figures 9, 10). Consequently, this delay helps to retain the information used in the past, specifically that resembling the queue structure. This characteristic contributes significantly to the high performance (Gallicchio et al., 2017) of the Deep-ESN.

In addition, to adapt the Deep-ESN to real-world data, the characteristics of its performance against time series involving stochastic noise must be considered. In Section 2 of the Supplementary material, the dependence of the NRMSE on the leaking rate is demonstrated against the time-series prediction of the sunspot time series, which involves stochastic behavior. Our results communicate that the estimation performance increases as the leaking rate approaches one. This suggests that suppressing the temporal history effect is essential for accurately modeling time-series data with stochastic noise, although this suppression may compromise the ability to capture long-term behaviors. To address this trade-off, we are currently exploring the integration of attention mechanisms into ESNs (Sakemi et al., 2024), which could further enhance the performance of Deep-ESNs for time-series data involving stochastic noise.

Although this study revealed the existence of an optimal leaking rate, a grid search is still necessary for a concrete set for the leaking rate in accordance with the tasks. This facilitates the need to develop an approach to determine the optimal leaking rate based on the dynamic characteristics used in this study. Moreover, many important benchmark tasks for evaluating deep neural networks have been introduced over the past decade. Notably, Moving Mixed National Institute of Standards and Technology database (MNIST) (Shi et al., 2015), motor imagery datasets (available at https://moabb.neurotechx.com/docs/datasets.html), and MLPerf (available at https://mlcommons.org/) serve as more contemporary standard benchmarks for deep neural networks. Therefore, it is essential to apply these datasets in addition to the classical prediction tasks described in our study to validate the capability of Deep-ESNs in practical scenarios. These points should be addressed in future studies.

## 5 Conclusion

In conclusion, through MSCE and cross-correlation analyses, this study has revealed the presence of an optimal leaking rate to represent the complex desired signal and a mechanism to retain past information in the Deep-ESN. Despite some limitations, these findings contribute to establishing optimum design guidelines for setting the hyperparameters of the Deep-ESN.

## Data availability statement

The original contributions presented in the study are included in the article/Supplementary material, further inquiries can be directed to the corresponding author.

## Author contributions

SI: Investigation, Methodology, Validation, Writing – original draft, Writing – review & editing. SN: Methodology, Validation, Writing – original draft, Writing – review & editing. HN: Investigation, Methodology, Validation, Writing – original draft, Writing – review & editing. EW: Methodology, Writing – review & editing. TI: Investigation, Methodology, Writing – review & editing.

## References

[B1] Adeleke O. A. (2019). “Echo-state networks for network traffic prediction,” in 2019 IEEE 10th Annual Information Technology, Electronics and Mobile Communication Conference (IEMCON) (New York, NY: IEEE), 202–206.

[B2] Adhikari A. Sigurdsson T. Topiwala M. A. Gordon J. A. (2010). Cross-correlation of instantaneous amplitudes of field potential oscillations: a straightforward method to estimate the directionality and lag between brain areas. J. Neurosci. Methods 191, 191–200. 10.1016/j.jneumeth.2010.06.019 20600317 PMC2924932

[B3] Bai Y.-T. Jia W. Jin X.-B. Su T.-L. Kong J.-L. Shi Z.-G. (2023). Nonstationary time series prediction based on deep echo state network tuned by Bayesian optimization. Mathematics 11:1503. 10.3390/math11061503

[B4] Bhandari A. (2017). Wavelets based multi-scale analysis of select global equity returns. Theor. Appl. Econ. 24:613.

[B5] Chen S. Shang P. (2020). Financial time series analysis using the relation between mpe and mwpe. Phys. A Stat. Mech. Appl. 537:122716. 10.1016/j.physa.2019.122716

[B6] Costa M. Goldberger A. L. Peng C.-K. (2002). Multiscale entropy analysis of complex physiologic time series. Phys. Rev. Lett. 89:068102. 10.1103/PhysRevLett.89.068102 12190613

[B7] Dean R. T. Dunsmuir W. T. (2016). Dangers and uses of cross-correlation in analyzing time series in perception, performance, movement, and neuroscience: the importance of constructing transfer function autoregressive models. Behav. Res. Methods 48, 783–802. 10.3758/s13428-015-0611-2 26100765

[B8] Deng L. Yu D. Platt J. (2012). “Scalable stacking and learning for building deep architectures,” in 2012 IEEE International Conference on Acoustics, Speech and Signal Processing (ICASSP) (New York, NY: IEEE), 2133–2136.

[B9] Gallicchio C. Micheli A. (2019). “Richness of deep echo state network dynamics,” in International Work-Conference on Artificial Neural Networks (Cham: Springer), 480–491.

[B10] Gallicchio C. Micheli A. (2021). Deep Reservoir Computing. Reservoir Computing: Theory, Physical Implementations, and Applications (Cham), 77–95.

[B11] Gallicchio C. Micheli A. Pedrelli L. (2017). Deep reservoir computing: a critical experimental analysis. Neurocomputing 268, 87–99. 10.1016/j.neucom.2016.12.089

[B12] Gallicchio C. Micheli A. Pedrelli L. (2018). Comparison between deepesns and gated rnns on multivariate time-series prediction. arXiv [preprint]. arXiv:1812.11527. 10.48550/arXiv.1812.11527

[B13] Glass L. Mackey M. (2010). Mackey-glass equation. Scholarpedia 5:6908. 10.4249/scholarpedia.6908

[B14] Humeau-Heurtier A. (2015). The multiscale entropy algorithm and its variants: a review. Entropy 17, 3110–3123. 10.3390/e17053110

[B15] Inoue S. Nobukawa S. Nishimura H. Watanabe E. Isokawa T. (2023). “Mechanism for enhancement of functionality in deep echo state network by optimizing leaking rate,” in 2023 International Conference on Emerging Techniques in Computational Intelligence (ICETCI) (New York, NY: IEEE), 85–90.

[B16] Jaeger H. (2001). The “Echo State” Approach to Analysing and Training Recurrent Neural Networks-With an Erratum Note. Bonn: German National Research Center for Information Technology GMD Technical Report 148, 13.

[B17] Jaeger H. (2007). Echo state network. Scholarpedia 2:2330. 10.4249/scholarpedia.2330

[B18] Jaeger H. Lukoševičius M. Popovici D. Siewert U. (2007). Optimization and applications of echo state networks with leaky-integrator neurons. Neural Netw. 20, 335–352. 10.1016/j.neunet.2007.04.016 17517495

[B19] Kanda K. Nobukawa S. (2022). “Feature extraction mechanism for each layer of deep echo state network,” in 2022 International Conference on Emerging Techniques in Computational Intelligence (ICETCI) (New York, NY: IEEE), 65–70.

[B20] Long J. Zhang S. Li C. (2019). Evolving deep echo state networks for intelligent fault diagnosis. IEEE Transact. Ind. Inf. 16, 4928–4937. 10.1109/TII.2019.2938884

[B21] Lukoševičius M. Jaeger H. (2009). Reservoir computing approaches to recurrent neural network training. Comp. Sci. Rev. 3, 127–149. 10.1016/j.cosrev.2009.03.005

[B22] Lukoševičius M. Uselis A. (2019). “Efficient cross-validation of echo state networks,” in Artificial Neural Networks and Machine Learning-ICANN 2019: Workshop and Special Sessions: 28th International Conference on Artificial Neural Networks, Munich, Germany, September 17-19, 2019, Proceedings 28 (Cham: Springer), 121–133.

[B23] Malik Z. K. Hussain A. Wu Q. J. (2016). Multilayered echo state machine: a novel architecture and algorithm. IEEE Trans. Cybern. 47, 946–959. 10.1109/TCYB.2016.2533545 27337730

[B24] Manneville P. Pomeau Y. (1979). Intermittency and the lorenz model. Phys. Lett. A 75, 1–2. 10.1016/0375-9601(79)90255-X

[B25] Rössler O. E. (1983). The chaotic hierarchy. Zeitschrift Naturforschung A 38, 788–801. 10.1515/zna-1983-0714

[B26] Sakemi Y. Nobukawa S. Matsuki T. Morie T. Aihara K. (2024). Learning reservoir dynamics with temporal self-modulation. Commun. Phys. 7:29. 10.1038/s42005-023-01500-w

[B27] Salehinejad H. Sankar S. Barfett J. Colak E. Valaee S. (2017). Recent advances in recurrent neural networks. arXiv [preprint]. arXiv:1801.01078. 10.48550/arXiv.1801.01078

[B28] Schrauwen B. Defour J. Verstraeten D. Van Campenhout J. (2007). “The introduction of time-scales in reservoir computing, applied to isolated digits recognition,” in Artificial Neural Networks-ICANN 2007: 17th International Conference, Porto, Portugal, September 9-13, 2007, Proceedings, Part I 17 (Cham: Springer), 471–479.

[B29] Shi X. Chen Z. Wang H. Yeung D.-Y. Wong W.-K. Woo W.-C. (2015). Convolutional lstm network: a machine learning approach for precipitation nowcasting. Adv. Neural Inf. Process. Syst. 28, 802–810. 10.5555/2969239.2969329

[B30] Tanaka G. Matsumori T. Yoshida H. Aihara K. (2022). Reservoir computing with diverse timescales for prediction of multiscale dynamics. Phys. Rev. Res. 4:L032014. 10.1103/PhysRevResearch.4.L032014

[B31] Tanaka G. Yamane T. Héroux J. B. Nakane R. Kanazawa N. Takeda S.. (2019). Recent advances in physical reservoir computing: a review. Neural Netw. 115, 100–123. 10.1016/j.neunet.2019.03.005 30981085

[B32] Tchakoucht T. A. Ezziyyani M. (2018). Multilayered echo-state machine: a novel architecture for efficient intrusion detection. IEEE Access 6, 72458–72468. 10.1109/ACCESS.2018.286734527337730

[B33] Venkatasubramanian V. Schattler H. Zaborsky J. (1995). Dynamics of large constrained nonlinear systems-a taxonomy theory [power system stability]. Proc. IEEE 83, 1530–1561. 10.1109/5.481633

[B34] Viehweg J. Worthmann K. Mäder P. (2023). Parameterizing echo state networks for multi-step time series prediction. Neurocomputing 522, 214–228. 10.1016/j.neucom.2022.11.044

[B35] Werbos P. J. (1990). Backpropagation through time: what it does and how to do it. Proc. IEEE 78, 1550–1560. 10.1109/5.58337

[B36] Williams R. J. Zipser D. (1989). A learning algorithm for continually running fully recurrent neural networks. Neural Comput. 1, 270–280. 10.1162/neco.1989.1.2.270

[B37] Yan B. He S. (2021). Dynamics and complexity analysis of the conformable fractional-order two-machine interconnected power system. Math. Methods Appl. Sci. 44, 2439–2454. 10.1002/mma.5937

