# Supplementary material for: Multi-scale dynamics by adjusting the leaking rate to enhance the performance of deep echo state networks
Source: Front Artif Intell. 2024 Jul 16;7:1397915. doi: 10.3389/frai.2024.1397915 (PMC11286403; doi:10.3389/frai.2024.1397915)
Supplement: Supplementary file 1 [file Data_Sheet_1.PDF]

Supplementary Material: Multi-scale Dynamics by Adjusting Leaking Rate to Enhance Performance of Deep Echo State Networks

1 MULTI-SCALE ENTROPY ANALYSIS FOR INPUT SIGNALS

The performance evaluation of the time-series prediction task revealed that the heterogeneous model was superior over the homogeneous model in the Mackey–Glass time-series task ($\tau = 64$). To investigate the reasons for this, multi-scale entropy (MSCE) analysis was applied to the Mackey–Glass, Lorenz, and Rössler time series. Figure S1 shows the dependence of SampEn on the time scale τ_s ($= 1, 2, \cdots, 20$) (left part) and the standard deviations among the time scales (right part). The results demonstrate that the complexity of the Mackey–Glass time series with $\tau = 64$ was more widely distributed on each time scale compared to the other signals, and this corresponds to the largest standard deviation compared to other signals. The heterogeneous model achieves a strong prediction ability against time series with such high multi-scalability.

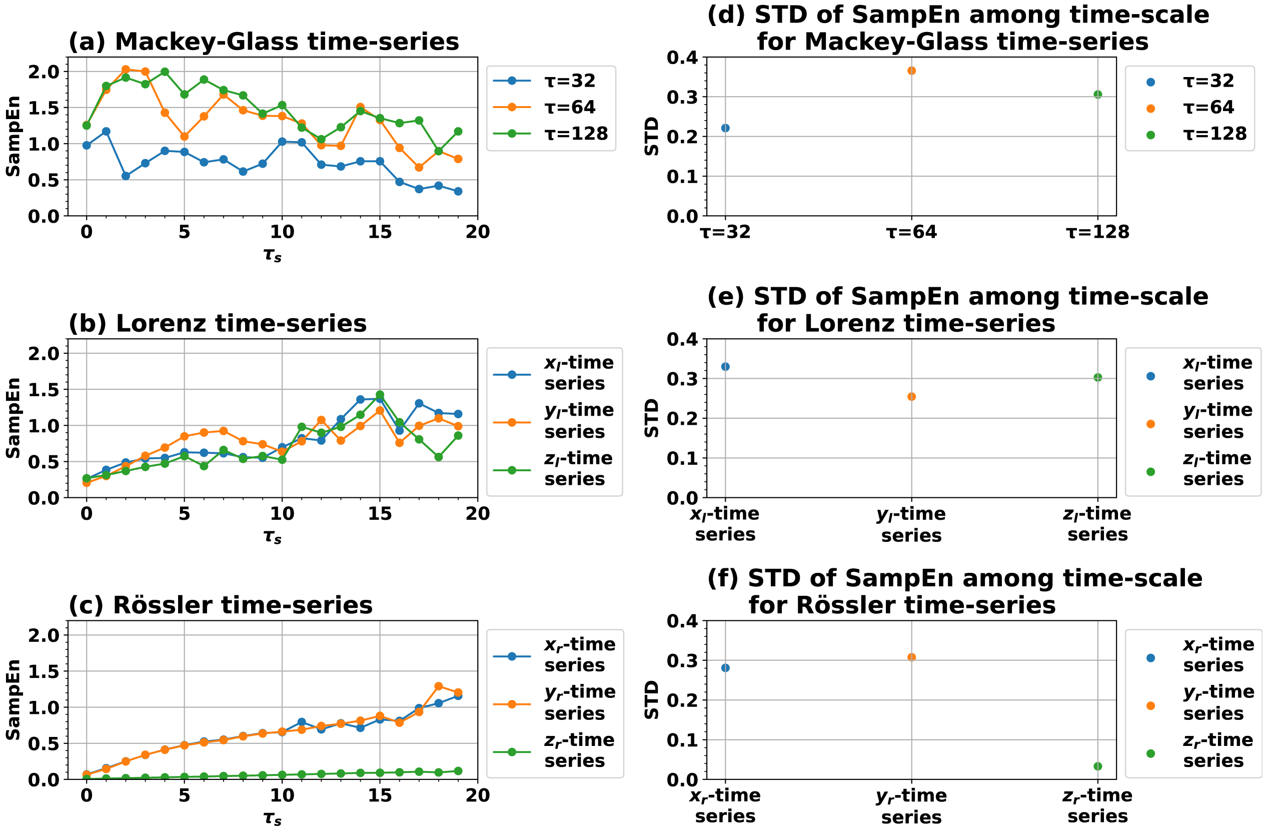

Figure S1. MSCE analysis for input signals. (Left panel) MSCE analysis of input signals from Mackey–Glass (a), Lorenz (b), and Rössler (c) time series across time-scale factors. $\tau_s = 1, 2, ..., 20$. (Right panel) Standard deviation (STD) of complexity (SampEn) among time scales. The Mackey–Glass time series ($\tau = 64$) exhibited the highest multi-scalability among the time series.

2 SUNSPOT TIME-SERIES PREDICTION TASK

In addition to predicting chaotic time-series tasks, we used sunspot time-series data from 1749 to August 2021, with a measuring interval of one month, as actual time series involving stochastic noise. Figure S2 depicts the dependence of the normalized root mean square error (NRMSE) on the leaking rate $a^{(l)}$ for five-step-ahead predictions using a homogeneous model with a spectral radius of $\rho = 0.9$ (the other parameters were the same as those in the main manuscript). We confirmed that the estimation performance increased as the leaking rate approached one. This suggests that suppressing the temporal history effect is essential for accurately estimating time-series data with stochastic noise.

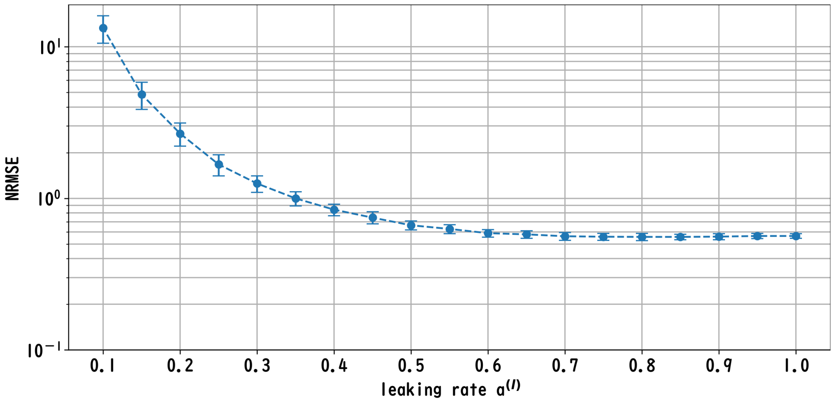

Figure S2. Dependence of the NRMSE on the leaking rate $a^{(l)}$ for five-step-ahead predictions of sunspot time series (`https://psl.noaa.gov/gcos_wgsp/Timeseries/SUNSPOT/`) using a homogeneous model with a spectral radius of $\rho = 0.9$. The results show that the estimation performance increased as the leaking rate approached one.

3 SIGNAL TRANSMISSIONS IN THE RESERVOIR STATE DYNAMICS AMONG LAYERS USING MUTUAL INFORMATION ANALYSIS

To evaluate the signal transmissions in the reservoir state dynamics among layers, in addition to cross-correlation, mutual information analysis is necessary to investigate the synchronization with delays in systems involving nonlinear dynamics. Figure S3 depicts the mutual information for the reservoir state dynamics between adjacent reservoir layers (the l-th and $l+1$-th layers): $\mathrm{MI}(k)$ in the case with the heterogeneous model (spectral radius $\rho = 1.0$) for the Mackey–Glass ($\tau = 64$) task. This setting corresponds to the highest accuracy in Fig. 3 (a) for the Mackey–Glass ($\tau = 64$) task. Lag k where the maximized $\mathrm{MI}(k)$ was achieved (represented by the red arrow) shows the signal transmission delay from the l-th to $l+1$-th layers. Similar to the results obtained with cross-correlation (Section 3.3 in the main manuscript), the mutual information peaked at a positive lag ($k > 0$), specifically between layers. This indicates that signal transmission delays also occurred from the l-th to $l+1$-th layers, considering the nonlinear relationships between the behaviors across layers.

4 CONTRIBUTION OF EACH LAYER TO PREDICTION TASKS

To evaluate the contribution of each layer to prediction tasks, we focus on the values of the components in $\mathbf{W}_{\mathrm{out}}$. If the absolute value of a component in $\mathbf{W}_{\mathrm{out}}$ is larger, this indicates that the neuron connected by this component makes a significant contribution. Figure 4 shows the components of $\mathbf{W}_{\mathrm{out}}$ corresponding

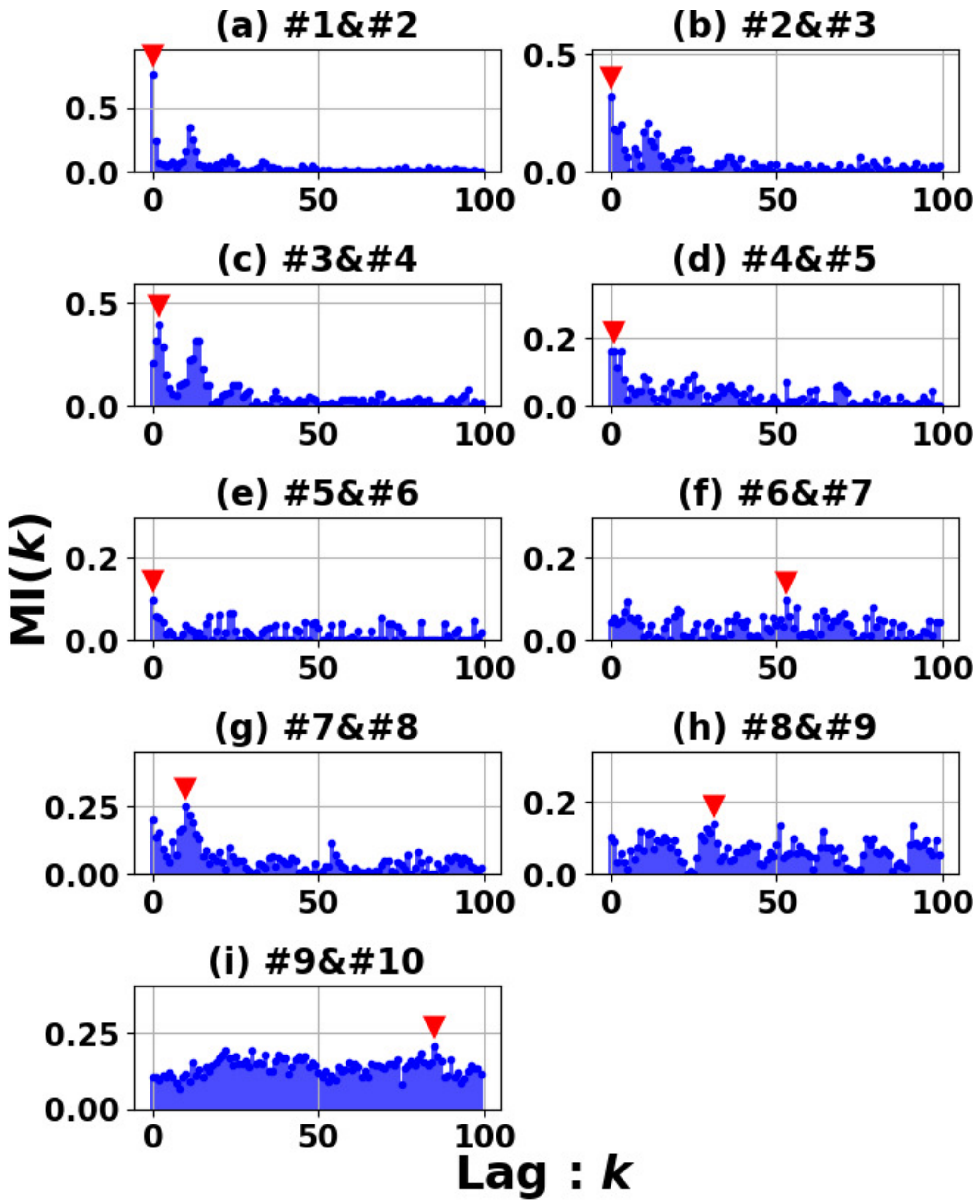

Figure S3. Mutual information for the dynamics of the reservoir states between adjacent reservoir layers (the l-th and $l+1$-th layers): MI(k) in the case with the heterogeneous model (spectral radius $\rho = 1.0$) for the Mackey–Glass ($\tau = 64$) task. This setting corresponds to the highest accuracy in Fig. 3 (a) for the Mackey–Glass ($\tau = 64$) task. Lag k where the maximized MI(k) was achieved (represented by the red arrow) shows the signal transmission delay from the l-th to $l+1$-th layers. In panels (c), (d), (f), (g), (h), and (i), the peaks are $k \geq 1$; hence, the reservoir dynamics were delayed between layers, considering the nonlinear relationships between the behaviors across layers.

to Fig. 3 (a) for the Mackey–Glass ($\tau = 64$) task over three trials. Each layer corresponds to $N_x = 100$ consecutive components. It can be observed that components with significantly large absolute values appeared in each layer. Therefore, the contributions to prediction tasks, which are facilitated by multi-scale behavior and layer-to-layer delays, were distributed among the layers.

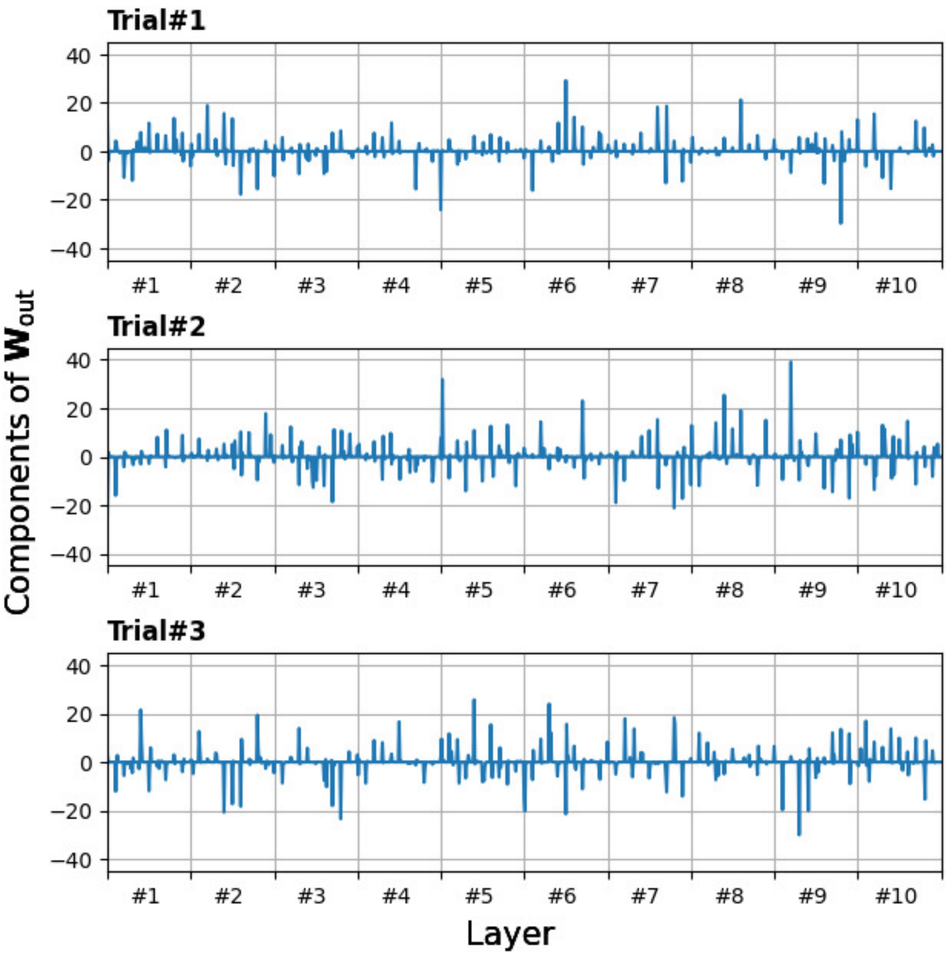

Figure S4. Components of $\mathbf{W}_{\text{out}}$ corresponding to Fig. 3 (a) for the Mackey–Glass task with $\tau = 64$, depicted across three trials. Each layer is represented by $N_x = 100$ consecutive components. Notably, components with significantly large absolute values were observed within each layer. These components are indicative of substantial contributions to the prediction tasks.
